# Rhinomodelation With Polycarpolactone—A Safer and Effective Solution for the Future

**DOI:** 10.1111/jocd.70001

**Published:** 2025-02-07

**Authors:** Kamran Izhar Qureshi, Franco Vercesi, Hina Farooq Qureshi

**Affiliations:** ^1^ Antiage Lifestyle Clinic Lahore Pakistan; ^2^ Centro Medico Galeno Milano Italy

**Keywords:** Ellanse, dermal filler, HA filler, Hyaluronic, hyaluronic acid, Non Surgical rhinoplasty, PCL Filler, rhinoplasty, semi‐permanent fillers

## Abstract

**Introduction:**

Nonsurgical rhinoplasty or rhinomodelation is a sought‐after procedure in aesthetic practice. The current product of choice remains hyaluronic acid (HA) because of its ease of use and reversibility. However, it does carry some risks. Polycaprolactone (PCL) fillers hold a lot of promise in aesthetic practice. It has an established safety profile and a longer duration of action. Because of its unique properties, it may hold the key to the future of nonsurgical rhinomodelation.

**Methods:**

Twelve patients were enrolled in the study. Ten out of these were not willing to ask for surgery and had no breathing problems. Two were post‐surgical rhinoplasty complications who did not want another surgery. 0.2 mL of PCL filler was injected at each site according to the need (radix, spine, and or tip). The Global Aesthetic Improvement Scale (GAIS; 3 = very much improved, 2 = considerably improved, 1 = improved, 0 = no change, and −1 = worse) was used along with a patient satisfaction scale (highly satisfied, satisfied, dissatisfied) were used. The sum of the GAIS ratings was quantified as total improvement (TMI). The patients were followed up for 12 months before and 12 months after procedures were taken and documented.

**Results:**

The GAIS score was 98% for the study and all patients were highly satisfied with their treatment right after procedure and 12 months later.

**Conclusion:**

PCL fillers may be the way forward for long‐term sustained results in nonsurgical rhinomodelation. Expert injection techniques and knowing the side effects and handling them is mandatory.

## 1 Introduction

Aesthetic surgery is one of the most evolving fields in today's era. According to the International Society of Aesthetic Plastic Surgery data 2023, Rhinoplasty is one of the most popular procedures with 1.1 million procedures and a 21.6% increase over the preceding year. In the nonsurgical procedures dermal fillers were 5.5 million with a 29% increase [1]. Surgical rhinoplasty though having over 80% success rate if performed by an experienced surgeon, still has the fear of going under a knife thus making nonsurgical rhinoplasty a sought‐after procedure [2].

Nonsurgical rhinoplasty (NSR) offers an alternative to traditional surgical nose procedures. It is a quick and efficient way of addressing minor defects in the shape of the nose. In the recent past, a lot of plastic surgeons have started using it post rhinoplasty to address the post‐surgical complications and expectations of patients. The use of nonsurgical techniques has gained popularity over time. As far as nonsurgical or medical rhinoplasty is concerned, the results are positive [3]. An important consideration is the patient satisfaction and long‐term complications like breathing difficulties [4]. The patient satisfaction towards nonsurgical rhinoplasty is good [5].

The traditional hyaluronic based fillers have been used for decades for nonsurgical rhinoplasty and have a good result. The problem with the HA fillers is that they have a limited lifespan. Another problem encountered is “Filler Migration.” Though under reported, it is possible that nose fillers placed at the bridge may spread laterally and make the nose look wider over time. Filler migration is usually the result of over‐ treating, poor technique, and incorrect product choice. The use of glasses can also push the filler downwards over time. Chae et al. [6] reported two sequential lumps due to migration of filler. One of these lumps was in the forehead despite being injected in the nose (confirmed by histopathology). Furthermore, it has been documented that filler migration can occur early or late after deep soft tissue and/or supraperiosteal HA administrations. Unwanted results in terms of appearance and functionality might result from this situation [7].

Polycaprolactone (PCL) is a collagen stimulator that holds a lot of potential in the field of aesthetics. With its unique property of stimulating collagen, it has lesser risk of migration. The bio stimulatory dermal fillers have proven efficacy and superiority to hyaluronic fillers in areas like the nasolabial folds [8]. It is proven through studies that PCL is completely excreted out of the body [9].

## 2 Materials and Methods

This study aimed to assess the efficacy and aesthetic outcomes of PCL filler, Ellansé (Sinclair Pharmaceuticals, London, UK) in nonsurgical rhinoplasty. Twelve patients who were fit for the nonsurgical approach were selected. Two cases out of the Twelve were of post‐surgical rhinoplasty complications and were not willing for a surgical intervention again. All the cases were those who refused the surgical approach and opted for nonsurgical rhinomodelation. The patients were in the age group 20–35. The patients were followed up for a period of 12 months to assess the aesthetic outcome and patient satisfaction. The enrollment period was December 2023.

### 2.1 Pre‐Treatment

Every patient underwent a thorough examination, which included a review of their medical history and a list of their current medications, before beginning treatment. The age group selected for the study was 18–50 years. Patients having a history of nasal problems were excluded of the study. Patients were asked about their allergies in general and allergy to hyaluronic or PCL fillers in particular. Detailed history was taken for cosmetic procedures. Patients having previous history of nonsurgical rhinoplasty were included in the study with a condition that the previous procedure should have been done at least 1 year ago. Patients in whom surgical rhinoplasty had failed and they were either not eligible or not willing for revised surgical intervention were included in the study. Patients suspected to be suffering from body dysmorphic disorder were excluded and so were patients with unrealistic expectations. To ascertain each patient's requirement retreatment photos of the nose were acquired. All participants signed and dated the study consent form and received patient information before beginning the study. Copies of the signed documents were given to the subjects, and the originals were stored in the subject's file. Ten patients out of the Twelve were reluctant to go for surgical rhinoplasty and opted for the nonsurgical option. Two patients were post‐rhinoplasty complications and were not willing for surgical revision again. patients had post‐rhinoplasty. The risks and benefits of using PCL were explained in detail to all the patients.

The Global Aesthetic Improvement Scale (GAIS; 3 = very much improved, 2 = considerably improved, 1 = improved, 0 = no change, and −1 = worse) was used to assess the outcome. The sum of the GAIS ratings was quantified as Total improvement (TMI). The GAIS was done by three independent evaluators (a dermatologist and two plastic surgeons) through photographic assessment. The photographs were taken before, after, and at 12 months after the procedure. The GAIS score was again calculated by comparing the pictures before and 12 months after the procedure.

The patients were asked to do a self‐assessment of the result through a questionnaire. They were asked if they were dissatisfied, satisfied, or highly satisfied (1 being highly satisfied and 3 being dissatisfied) right after the procedure and on 12th month when they came for follow‐up.

### 2.2 Injection Technique

The same medical professional (dermatologist in this case) used the same filler for each injection, Ellanse M in these cases. The procedure started with the application of a topical anesthetic to the nasal region, followed by a 15‐min wait. To begin the process, the target location was first made numb and then cleaned using an antiseptic fluid.

The PCL filler was injected by the prefilled syringe provided by the manufacturer. The needle used for the injection was 27 G × ¾″ manufactured by Terumo, Europe provided and recommended by the filler manufacturer.

Two nasal regions—the radix and nasal dorsum were given PCL filler injections based on the patient's demands. When employing the injection technique, a maximum of 0.2 cc of PCL was administered at each injection site. Supra periosteal for the radix and dorsum.

Total maximum of 0.4 mL of PCL filler was injected.

- 0.1–0.2 mL bolus of the PCL filler was injected perpendicularly to the level of the periosteum.
- Further if needed two boluses were injected at 3 mm from the first one in the cranio‐caudal direction.

### 2.3 Post Procedure Care

A steri‐strip was placed on both sides of the nose for a period of 2 days. The patients were advised not to massage the area and not to wear any glasses for a period of 2–3 weeks. They were explained that redness and swelling in the injected area may be there. Patients were advised to take tablet Paracetamol for the pain. Patients were asked to refrain from ibuprofen and aspirin because of the chance of increase in bruising. They were further advised to contact in case of pain that was not resolved by pain killer, excessive swelling, or any skin change in the adjacent area. Further instructions included no massaging the area, sun and heat exposure, smoking, alcohol, sauna, and swimming for the first 24 h. Patients were recommended to refrain from wearing sunglasses, goggles, or reading glasses for a period of 15 days to avoid pressure on the injected area.

## 3 Results

**FIGURE 1:**
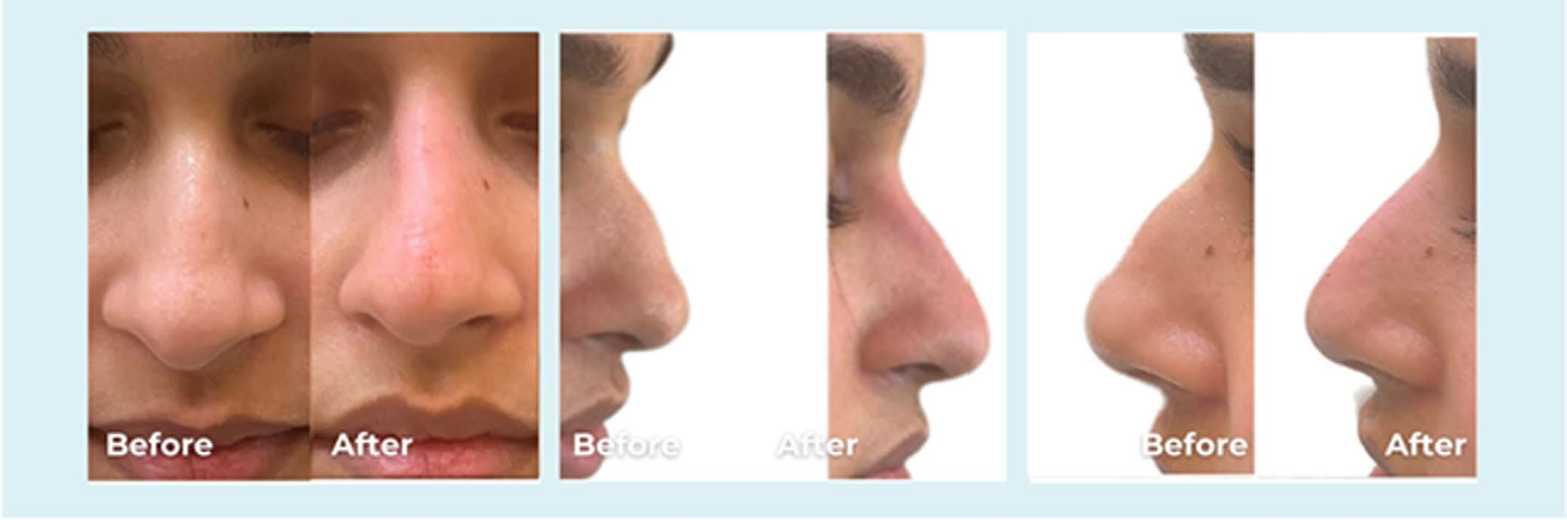
Frontal and lateral views of a 25‐year old female. The dorsal hump corrected by injecting 0.2 mL PCL filler into the radix above and 0.2 mL below the hump to straighten the profile.

**FIGURE 2:**
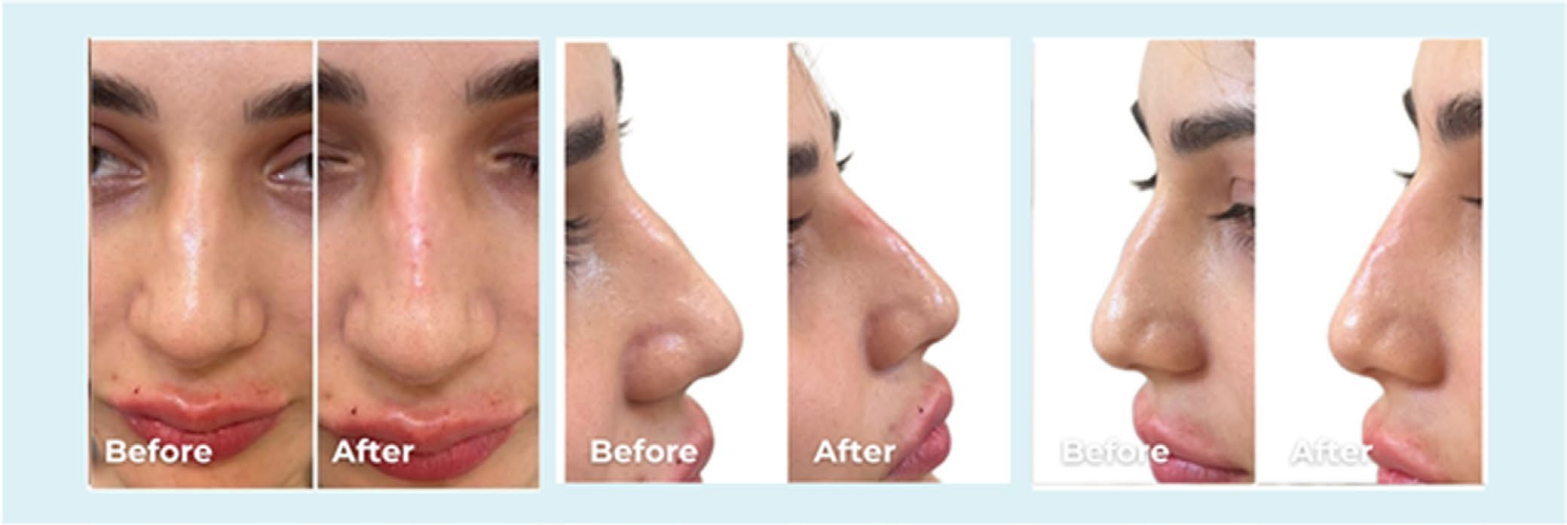
Frontal and lateral views of a 25‐year old female. The dorsal hump corrected by injecting 0.2 mL PCL filler into the radix above and 0.1 mL below the hump to straighten the profile.

**FIGURE 3:**
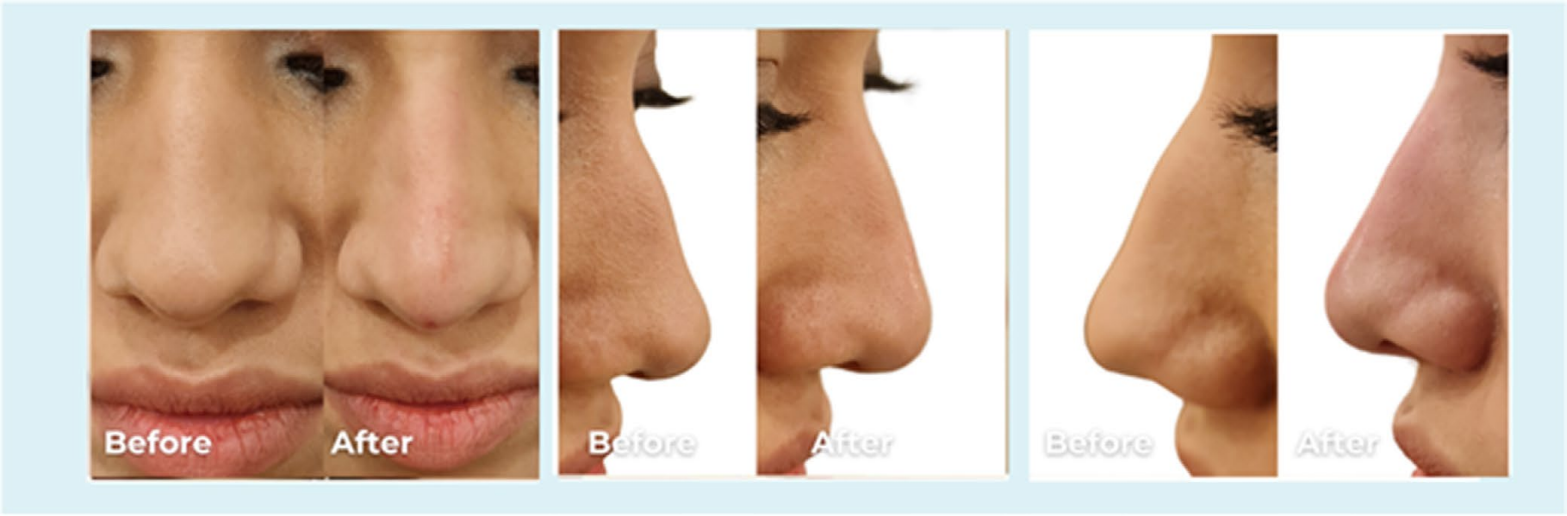
Frontal and lateral views of a 28‐year‐old female. The dorsal hump corrected by injecting 0.1 mL PCL filler into the radix above and 0.1 mL below the hump to straighten the profile, and 0.1 mL to raise the tip.

**FIGURE 4:**
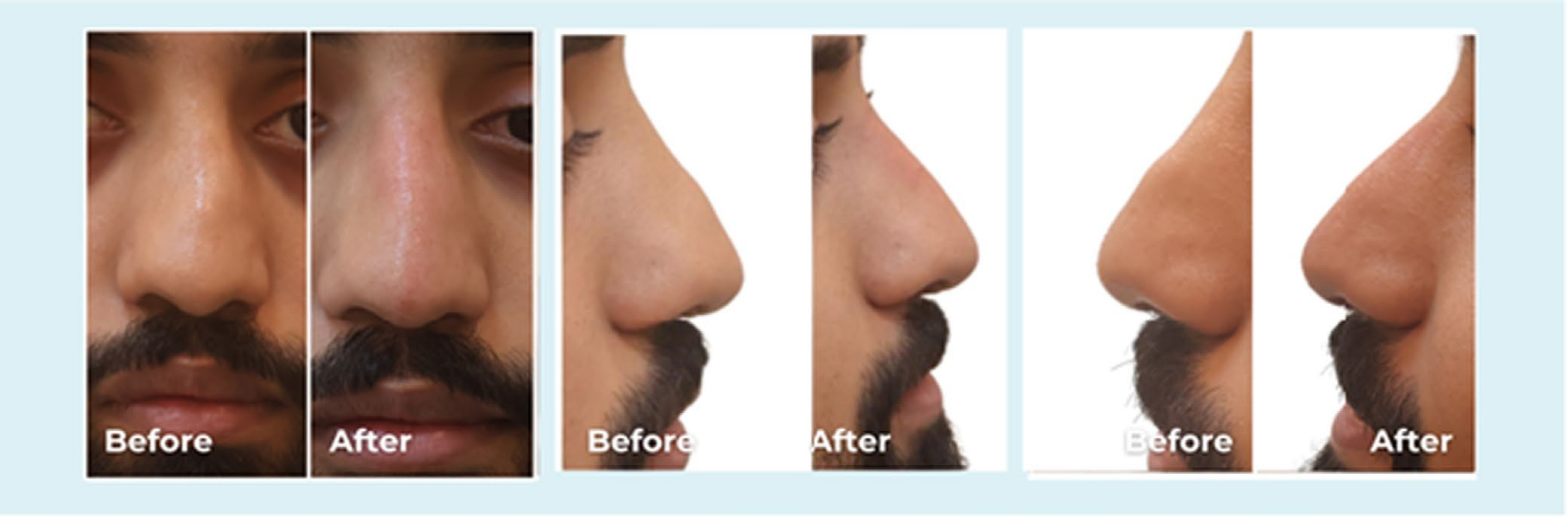
Frontal and lateral views of a 27‐year‐old male. The patient had a history of two previous rhinoplasties with a graft, but the results were not aesthetically pleasing. 0.2 mL PCL was injected as a spreader graft on the deviated side, 0.2 mL was injected to improve the NF angle, and 0.2 mL in the supratip area to achieve the aesthetic outcome.

**FIGURE 5:**
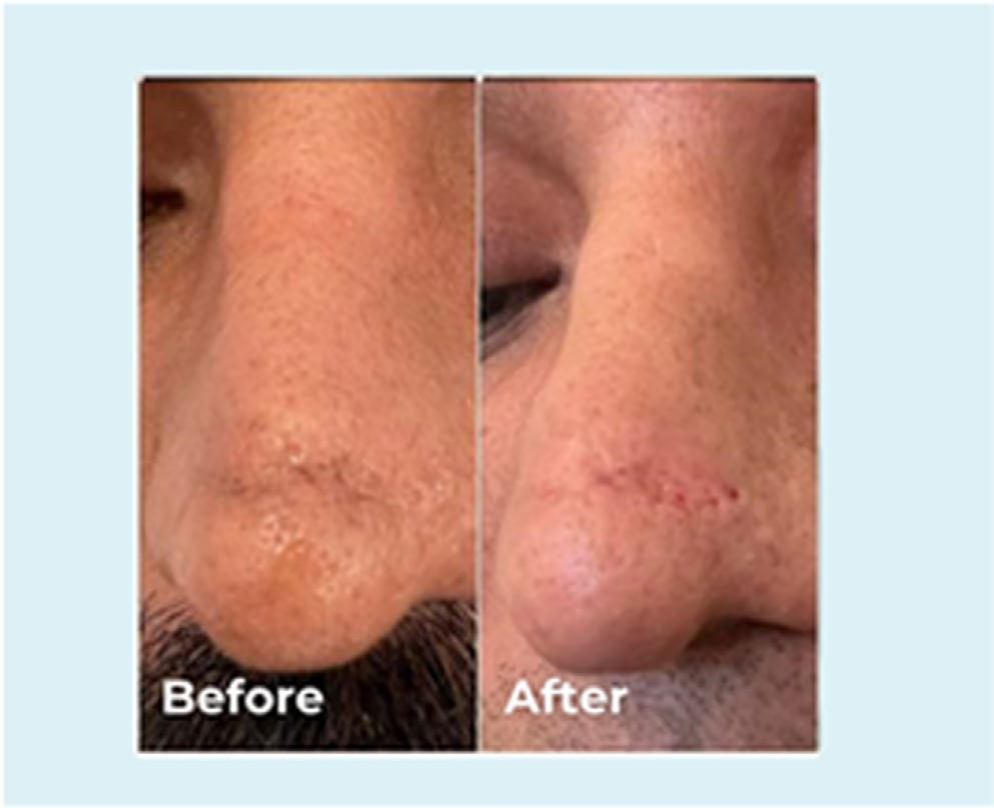
Frontal view of a 35‐year‐old male. The patient had a scar post rhinoplasty. 0.2 mL PCL filler into the scar to reduce its appearance.

**FIGURE 6:**
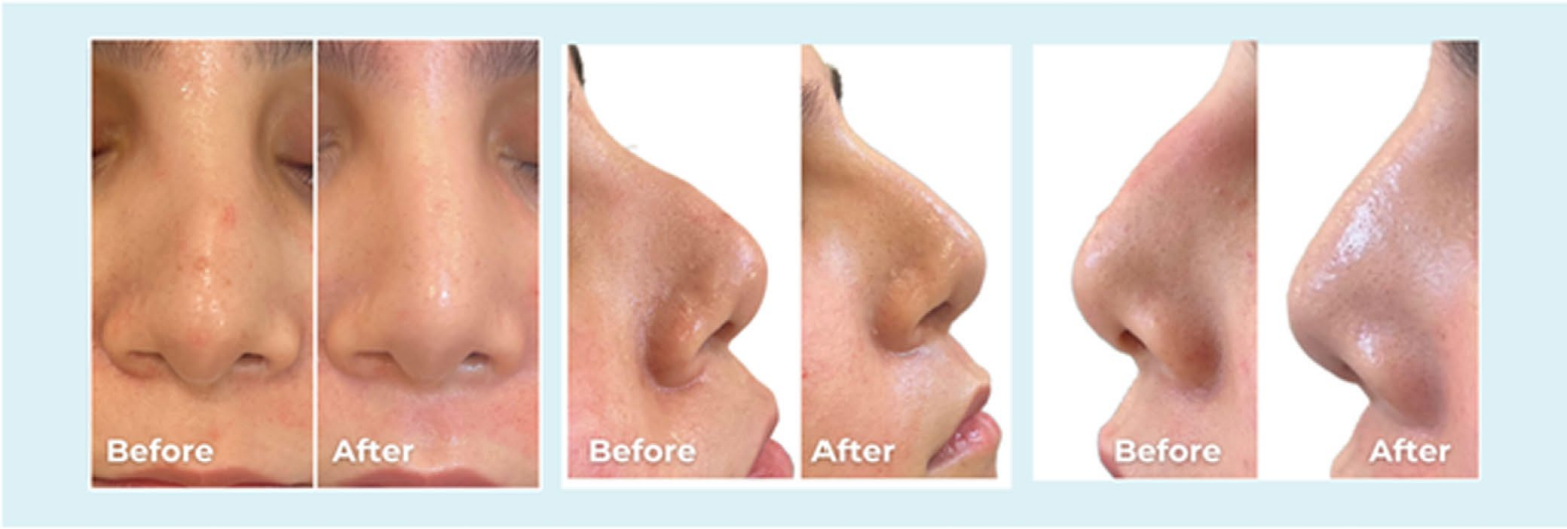
Frontal and lateral views of a 29‐year‐old female. The dorsal hump corrected by injecting 0.2 mL PCL filler into the radix above and 0.1 mL below the hump to straighten the profile.

Results of this study demonstrate that medical remodeling of the nose using a PCL filler is a safe, reliable technique with high patient satisfaction (Tables 1, 2, 3 and Figures 1, 2, 3, 4, 5, 6).

**TABLE 1:** Global aesthetic improvement scale (GAIS) score.

GAIS score			GAIS score		GAIS score	
First evaluator			Second evaluator		Third evaluator	
Patient no.	Right after procedure	12 months after procedure	Right after procedure	12 months after procedure	Right after procedure	12 months after procedure
1	2	2	2	3	1	2
2	2	2	2	2	2	2
3	2	3	2	3	2	3
4	3	3	2	3	3	3
5	2	2	2	2	2	2
6	2	3	3	3	2	3
7	3	3	3	3	3	3
8	2	2	1	2	3	3
9	2	3	3	3	2	2
10	3	3	3	3	3	3
11	3	3	2	2	3	3
12	2	3	3	3	3	3
Total	28	32	28	32	29	32

In the current study GAIS evaluations was done by three independent evaluators. Two of these were plastic surgeons and one a dermatologist. The assessment of all three was 90% for the treated patient after 12 months follow‐up. Right after the procedure the assessment was 80% (Table 1).

The patient assessment questionnaire revealed that all patients were very satisfied with their results both post right after the procedure and 12 months after the procedure (Tables 2 and 3).

**TABLE 2:** Patient self‐assessment score.

Patient self‐assessment score			
Patient	Right after procedure	6 months after procedure	12 months after procedure
1	1	1	1
2	1	1	1
3	1	1	1
4	1	1	1
5	1	1	1
6	1	1	1
7	1	1	1
8	1	2	1
9	1	1	1
10	1	1	1
11	1	1	1
12	1	1	1

**TABLE 3:** Patient assessment score.

Patient assessment score		
Patient	Right after procedure	12 months after procedure
1	Highly satisfied	Highly satisfied
2	Highly satisfied	Highly satisfied
3	Highly satisfied	Highly satisfied
4	Highly satisfied	Highly satisfied
5	Highly satisfied	Highly satisfied
6	Highly satisfied	Highly satisfied
7	Highly satisfied	Highly satisfied
8	Highly satisfied	Highly satisfied
9	Highly satisfied	Highly satisfied
10	Highly satisfied	Highly satisfied
11	Highly satisfied	Highly satisfied
12	Highly satisfied	Highly satisfied

Volume range of the PCL filler injected was between 0.2 and 0.4 mL. The exact quantity injected is shown in Figures 1, 2, 3, 4, 5, 6. In all the cases PCL was placed deeply, over bony, and cartilage tissue, just above the periosteum and/or the perichondrium; this was done to avoid vessels cannulation and related vascular problems.

## 4 Discussion

Heden et al. [10] did an analysis of over 250 individuals who had HA treatment for nose contouring since 1997. Moreover, HA injection effectively treated nasal deformities that would have been challenging to fix surgically, serving as a supplement to surgery. With some patients experiencing longer durations, the effect lasted for more than a year (up to 5 years).

Presently available on the market are a variety of fillers, each with unique properties. But aside from HA or other options, we think PCL is a good choice with clear benefits in several areas and indications. It should be mentioned that most HA products—especially the ones that stay longer—are made of highly reticulated HA. There is still much to learn about these devices' long‐term toxicity [11].

The reason for choosing polycaprolactone (PCL) is that it has a biocompatible, biodegradable, bioresorbable, and original cellular response polymer demonstrating long‐term efficacy and duration of action [12]. It has been shown that PCL is completely excreted from the body. It is used in daily clinical practice and prevention and treatment recommendations are well defined both through evidence and experience. First synthesized in the 1930s, there is enough evidence in favor of its better viscoelastic properties than other biodegradable polymers [13]. The design of the filler has shown a better safety profile and longer lasting effect than collagens and free hyaluronic acids. In comparison to Sculptra (Poly L‐Lactic Acid) (Galderma Laboratories, USA) and Radidesse (Calcium Hydroxapetite) (Merz North America, USA), the PCL‐based collagen stimulator, Ellansé, has the longest action duration [14]. It has been used for subdermal implantation for long‐lasting facial wrinkle correction and hand rejuvenation, since approval in 2009. The PCL filler is composed of regular PCL round‐microspheres suspended in a tailor‐made aqueous CMC gel carrier.

Both immunohistochemistry studies and quantitative and qualitative studies using Vectra 3D have shown that the effect of PCL fillers may last up to a period of 2 years [14, 15].

On the other hand CAHA based fillers have shown limited clinical efficacies. Studies show a 6–24 months effect which brings a considerable doubt towards the long‐term efficacy [16, 17].

### 4.1 PCL Resorption Mechanism

When thinking about biopolymers, it's crucial to remember that anything that is biodegradable does not always mean that it is bioresorbable; in other words, just because something breaks down and travels away from its site of action in vivo, it does not always mean that the body gets rid of it. On the other hand, the idea of bioresorbability refers to the complete removal of the original foreign materials and bulk breakdown products by‐products (low molecular weight molecules) with no lingering adverse effects [18].

When water enters the microspheres, the ester linkages throughout the whole polymer matrix gradually hydrolyze, leading to a bulk degradation process that is characteristic of PCL‐filler degradation by hydrolysis. First, the mass, volume, and form of the implant stay constant over time, but the length and molecular weight of the polymer chain drop. Next, diffusion of the tiny polymer fragments occurs after hydrolysis has created low molecular weight chains. In the microspheres, PCL shows both amorphous and crystalline parts; the amorphous portions are more readily hydrolyzed than the crystalline regions. Longevity of the microspheres ultimately depends on the hydrolytic disintegration of PCL crystalline areas. The chain length (molecular weight) of the Ellansé range items is what sets them apart. The PCL monomers (6‐hydroxycaproic acid) undergo a series of reactions that end in the TCA cycle [18].

### 4.2 PCL Mechanism of Action

The two components of the filler are CMC and PCL. The PCl microspheres are suspended in a customized aqueous CMC gel carrier. The CMC gel leads to an immediate effect and the PCL leads to a sustained effect [14]. The sustained effect is sustained by collagen production and the 3D‐scaffold formed, preventing cluster formation and prolonging the effect.

Collagen, the most predominant protein in the human body and skin, plays a crucial role in the extracellular matrix (ECM), and skin changes. The PCL filler, a collagen stimulator, has been shown to increase collagen production in both animals and humans hence, inducing a tissue repair process through inflammation, proliferation, and remodeling [19].

The long‐term efficacy and duration of action has been shown through clinical studies. It has been proven that a PCL‐based collagen stimulator can improve facial volume, forehead augmentation, and skin quality [15]. The long‐term efficacy of the PCL filler is demonstrated in volume restoration, contour redefinition, skin rejuvenation, skin quality, and wrinkle reduction [20, 21]. Galadari et al. [22] in their study showed the superiority of PCL fillers over HA fillers over the period of 12 months. A randomized, split faced study clearly concluded in favor of PCL‐based dermal fillers.

### 4.3 PCL Safety Profile

Clinical studies have shown that the PCL filler has shown no serious adverse events globally. No granuloma or vascular complications has been reported. However, like all other fillers there may be some injection related responses. These being mainly edema or ecchymosis, which are usually mild and resolved within a few days without intervention [23]. In a European clinical study, injection site reactions were similar to any filler, with swelling and bruising disappearing in 2–4 days. The long‐term safety of the PCL filler was confirmed in a clinical study following safety up to 30 months [24].

From 2009 to December 2017, 355 adverse events were reported, with a low adverse event rate of 0.056% [25]. A review from launch to December 2020 found the adverse event rate to be 0.0462% or 1% in 2615 syringes [19]. The overall safety profile of the PCL‐based collagen stimulator is good. However, there have been cases of late reactions, granuloma, discoloration, and xanthelasma‐like reactions. A review of complications in Korea showed good safety in 780 treated subjects from April 2015 to May 2018, with edema and bruising being the most common [26]. Overall, the PCL filler has shown good long‐ term efficacy, long action duration, and safety in aesthetic treatment.

### 4.4 Complication Prevention and Treatment: Recommendations

Physicians must be aware of the product characteristics to avoid adverse events. An important consideration should be given to right patient selection, proper aseptic conditions, and the right injection technique [27]. Preventing and managing complications of PCL fillers is essential for optimal aesthetic outcomes. Following strict rules and general recommendations is crucial for PCL‐based collagen stimulator treatment. Prevention measures have been discussed in various articles and expert reviews [8].

### 4.5 Prevention of Adverse Events

- Pre‐procedural, post‐procedural, and post‐procedural care
- Proper selection of patients
- Thorough patient assessment, understanding of expectations, and aesthetic assessment
- Right product selection
- Strict adherence to Instructions for Use (IFU)
- Aseptic conditions to prevent infection
- Recognized and trained physicians should inject
- Knowing and focusing on anatomy, product characteristics, and injection techniques. Proper advice on post‐procedural care

### 4.6 Management of Adverse Event: Recommendations

Dermal fillers are a common treatment option for a variety of conditions, but severe adverse events are rare. To manage these effects, physicians must localize and recognize the culprit product. Knowing the anatomy and blood supply of any area to be injected is mandatory [28]. It is necessary to rule out whether the PCL filler is causing the event or not. The injected product, whether PCL or HA filler or any other product should be considered Guilty until proven innocent. Recent addition of ultrasound in aesthetics, can help identify the filler, improve diagnosis, and guide treatment. The most common minor side effects are swelling/edema and the rare ones are nodules/lumps and granulomas.

Christen et al. [19] in their study have provided a comprehensive guideline to manage complications with PCL fillers. Swelling/edema is a normal inflammatory reaction to the trauma caused by the injection or large volume injected, which should disappear within 5–7 days. Prophylaxis with anti‐inflammatory enzymes, arnica/gelsemium, cold compresses, and anti‐inflammatory drugs are recommended for moderate cases. Persistent edema localized at the treated zone that lasts more than 7 days until 2 weeks is logically based on oral corticosteroids. For dermal fillers in general, long‐lasting malar edema responds poorly to treatment, and experts recommend prevention.

Nodules should not be confused with granulomas, as they are noninflammatory and hard, localized at the injection sites, pea‐shaped, and not increasing in size. Treatment depends on the time of onset and a wait and see attitude is recommended. Nodules/lumps occurring early after injection are generally related to a technical error, and intralesional microinjection of corticosteroids is the standard treatment. Treatment generally needs to be repeated.

Inflammatory nodules/granuloma are rare but severe adverse events that can occur 6–24 months after injection. They are a secondary late‐onset chronic inflammatory reaction with varying etiology, occurring 6–24 months after injection. Treatment is based on intralesional corticosteroids (high‐dose triamcinolone mixed with lidocaine and 5‐fluorouracil) to prevent recurrence and skin atrophy. Oral corticosteroids are often associated with recurrent granuloma, but surgical therapy is a last resort due to the difficulty of removing the granuloma completely and the risk of infection and scars.

The PCL filler is not known to be associated with granuloma, but it is important to provide information on the treatment recommended by a group of experts. The treatment described in detail starts by systemic treatment with prednisone 1 mg/kg per day for 1 week with intralesional injection of microinjection of a solution of corticosteroid, methylprednisolone or triamcinolone 20 mg/mL final concentration; methotrexate or 5‐FU can be added. This can be a long‐term treatment of several months, which can be stopped and reinitiated according to progression.

A new technique for nodules/granuloma treatment was developed in recent years as an option before surgery: the intraalesional laser treatment (ILT). This technique applied to the PCL filler showed benefit in the very few treated cases due to the extremely low AE incidence. The physico‐chemical properties of the PCL polymer, with a low melting point, should make it particularly sensitive to this technique.

Nose is an area of high vascularity. Complications can be both intravascular and/or pressure effect leading to occlusion. Knowing the anatomy is of utmost importance. Deep injection at the level of the supraperiostem is recommended and a bolus not more than 0.2 mL at each site is recommended. On the dorsum, center of the nose injection is a safe way to avoid vascular compromise. Under correcting the area is another key factor for consideration. Early bumps if developed can be controlled with massage. In case of a delayed complication the above guidelines are of utmost importance while handling them. Surgical intervention may be needed if the problem persists.

## 5 Conclusion

PCL fillers hold a lot of promise because of their collagen stimulating effect that may result in long‐term sustained results. The use of PCL in nonsurgical rhinomodelation has not been studied. Expert injection techniques are of highest importance and knowledge of handling the adverse events if any are of utmost consequence.

In this study a high satisfaction assessment score was achieved and there were no reported major complications. The study outcomes clearly confirm that injecting PCL filler into the deep planes can lead to an aesthetically pleasing results with high patient satisfaction. The study had the limitation of being an open label and had less patients but larger, randomized trials in future could lead to strengthening the current results. Though done for a follow‐up period of 9 months, based on the data available for PCL fillers we can conclude that the results will be long lasting.

## Author Contributions

All authors contributed to data analysis, drafting or revising the article, gave final approval of the version to be published, and agree to be accountable for all aspects of the work.

## Disclosure

Two authors are consultants for Sinclair Pharma. Dr. Kamran Izhar Qureshi is a trainer for Sinclair Pharma and Dr. Franco Vercesi is the International Key Opinion Leader for Sinclair Pharma. Dr. Hina Farooq Qureshi has no association with Sinclair Pharma.

## Conflicts of Interest

The authors declare no conflicts of interest.

## Data Availability

The data that support the findings of this study are available on request from the corresponding author. The data are not publicly available due to privacy or ethical restrictions.

## References

[1] International Society of Aesthetic Plastic Surgery , “ISAPS International Survey on Aesthetic/Cosmetic Procedures,” accessed November 17, 2024, https://www.isaps.org/media/rxnfqibn/isaps‐global‐survey_2023.pdf.

[2] N. Beneduce , C. Botter , E. Coiante , B. Hersant , and J. P. Meningaud , “The Longevity of the Nonsurgical Rhinoplasty: A Literature Review,” Journal of Stomatology Oral and Maxillofacial Surgery 124, no. 1S (2023): 101319, 10.1016/j.jormas.2022.10.018. 36280110

[3] D. S. Al‐Taie , E. M. AlEdani , J. Gurramkonda , et al., “Non‐Surgical Rhinoplasty (NSR): A Systematic Review of Its Techniques, Outcomes, and Patient Satisfaction,” Cureus 15, no. 12 (2023): e50728, 10.7759/cureus.50728. 38234960 PMC10792339

[4] G. Giammarioli and A. Liberti , “Non‐Surgical Rhinoplasty Technique: An Innovative Approach for Nasal Reshaping With Hyaluronic Acid Fillers,” Journal of Cosmetic Dermatology 22, no. 7 (2023): 2054–2062, 10.1111/jocd.15669. 36751855

[5] L. Di Rosa , G. Cerulli , and A. De Pasquale , “Psychological Analysis of Non‐Surgical Rhinoplasty,” Aesthetic Plastic Surgery 44, no. 1 (2020): 131–138, 10.1007/s00266-019-01538-8. 31768580

[6] S. Y. Chae , K. C. Lee , Y. H. Jang , S. J. Lee , D. W. Kim , and W. J. Lee , “A Case of the Migration of Hyaluronic Acid Filler From Nose to Forehead Occurring as Two Sequential Soft Lumps,” Annals of Dermatology 28, no. 5 (2016): 645–647, 10.5021/ad.2016.28.5.645. 27746650 PMC5064200

[7] S. Hamed‐Azzam , C. Burkat , A. Mukari , et al., “Filler Migration to the Orbit,” Aesthetic Surgery Journal 41, no. 6 (2021): NP559–NP566, 10.1093/asj/sjaa264. 32887989

[8] F. de Melo , P. Nicolau , L. Piovano , et al., “Recommendations for Volume Augmentation and Rejuvenation of the Face and Hands With the New Generation Polycaprolactone‐Based Collagen Stimulator (Ellansé®),” Clinical, Cosmetic and Investigational Dermatology 10 (2017): 431–440, 10.2147/CCID.S145195. 29184426 PMC5685142

[9] M. A. Woodruff and D. W. Hutmacher , “The Return of a Forgotten Polymer—Polycaprolactone in the 21st Century,” Progress in Polymer Science 35 (2010): 1217–1256.

[10] P. Hedén , “Nasal Reshaping With Hyaluronic Acid: An Alternative or Complement to Surgery,” Plastic and Reconstructive Surgery. Global Open 4, no. 11 (2016): e1120, 10.1097/GOX.0000000000001120. 27975025 PMC5142491

[11] J. Fidalgo , P. A. Deglesne , R. Arroyo , L. Sepúlveda , E. Ranneva , and P. Deprez , “Detection of a New Reaction By‐Product in BDDE Cross‐Linked Autoclaved Hyaluronic Acid Hydrogels by LC‐MS Analysis,” Medical Devices 11 (2018): 367–376, 10.2147/MDER.S166999. 30410412 PMC6197218

[12] H. Sun , L. Mei , C. Song , X. Cui , and P. Wang , “The In Vivo Degradation, Absorption, and Excretion of PCL‐Based Implant,” Biomaterials 27, no. 9 (2006): 1735–1740, 10.1016/j.biomaterials.2005.09.019. 16198413

[13] S. C. Woodward , P. S. Brewer , F. Moatamed , A. Schindler , and C. G. Pitt , “The Intracellular Degradation of Poly(Epsilon‐Caprolactone),” Journal of Biomedical Materials Research 19, no. 4 (1985): 437–444, 10.1002/jbm.820190408. 4055826

[14] M. Angelo‐Khattar , “Objective Assessment of the Long‐Term Volumizing Action of a Polycaprolactone‐Based Filler,” Clinical, Cosmetic and Investigational Dermatology 28, no. 15 (2022): 2895–2901, 10.2147/CCID.S385202. PMC9805706 36597519

[15] M. M. Moers‐Carpi and S. Sherwood , “Polycaprolactone for the Correction of Nasolabial Folds: A 24‐Month, Prospective, Randomized, Controlled Clinical Trial,” Dermatologic Surgery 39, no. 3 Pt 1 (2013): 457–463, 10.1111/dsu.12054. 23350617 PMC3615178

[16] F. Simunovic , S. Schlager , M. Montanari , and N. Iblher , “Prospective 3D Analysis of Facial Soft Tissue Augmentation With Calcium Hydroxylapatite,” Journal of Cosmetic and Laser Therapy 19, no. 5 (2017): 283–289, 10.1080/14764172.2017.1307411. 28328289

[17] M. Angelo‐Khattar , “Objective Evaluation of the Longevity of a Calcium Hydroxylapatite‐Based Filler (Radiesse),” Journal of Clinical and Cosmetic Dermatology 5, no. 2 (2021): 1–5.

[18] A. S. Turaev , “Dependence of the Biodegradability of Carboxymethylcellulose on Its Supermolecular Structure and Molecular Parameters,” Chemistry of Natural Compounds 31 (1995): 254–259.

[19] M. O. Christen and F. Vercesi , “Polycaprolactone: How a Well‐Known and Futuristic Polymer has Become an Innovative Collagen‐Stimulator in Esthetics,” Clinical, Cosmetic and Investigational Dermatology 13 (2020): 31–48, 10.2147/CCID.S229054. 32161484 PMC7065466

[20] J. Varani , M. K. Dame , L. Rittie , et al., “Decreased Collagen Production in Chronologically Aged Skin: Roles of Age‐ Dependent Alteration in Fibroblast Function and Defective Mechanical Stimulation,” American Journal of Pathology 168, no. 6 (2006): 1861–1868, 10.2353/ajpath.2006.051302. 16723701 PMC1606623

[21] J. A. Kim and D. Van Abel , “Neocollagenesis in Human Tissue Injected With a Polycaprolactone‐Based Dermal Filler,” Journal of Cosmetic and Laser Therapy 17, no. 2 (2015): 99–101, 10.3109/14764172.2014.968586. 25260139

[22] H. Galadari , D. van Abel , K. Al Nuami , F. Al Faresi , and I. Galadari , “A Randomized, Prospective, Blinded, Split‐Face, Single‐Center Study Comparing Polycaprolactone to Hyaluronic Acid for Treatment of Nasolabial Folds,” Journal of Cosmetic Dermatology 14, no. 1 (2015): 27–32, 10.1111/jocd.12126. 25564797

[23] S. Lin , “Complications of a PCl‐Based Dermal Filler: Causes, Management, Prevention and Incidence Rate,” Communication at IMCAS Americas (Cartagena de Indias: Google Scholar, 2019).

[24] M. O. Christen and J. Meadows , “Ellansé: Science and Safety Update,” (2018), Communication at the World Expert Meeting (WEM). Barcelona.

[25] A. Sheikh and J. Smith , “Ellanse® Safety: PMS,” Sinclair Internal Data (France: Sinclair Pharma, 2018).

[26] S. L. Lin and M. O. Christen , “Polycaprolactone‐Based Dermal Filler Complications: A Retrospective Study of 1111 Treatments,” Journal of Cosmetic Dermatology 19, no. 8 (2020): 1907–1914, 10.1111/jocd.13518. 32485052 PMC7497126

[27] B. Baser , P. Singh , P. Shubha , P. K. Roy , and P. Chaubey , “Non‐Surgical Rhinoplasty and Use of Hyaluronic Acid Based Dermal Filler‐User Experience in Few Subjects,” Indian Journal of Otolaryngology and Head & Neck Surgery 73, no. 1 (2021): 52–58, 10.1007/s12070-020-02100-8. 33643885 PMC7881997

[28] S. M. Lam and E. F. Williams, 3rd. , “Anatomic Considerations in Aesthetic Rhinoplasty,” Facial Plastic Surgery 18, no. 4 (2002): 209–214, 10.1055/s-2002-36488. 12524592

